# Asymmetric Bilateral Axenfeld-Rieger Anomaly Misdiagnosed as Unilateral Iridocorneal Endothelial Syndrome

**DOI:** 10.7759/cureus.115031

**Published:** 2026-08-23

**Authors:** Nishi Satish, Saanchal Sethi

**Affiliations:** 1 Ophthalmology, Vardhman Mahavir Medical College and Safdarjung Hospital, New Delhi, IND

**Keywords:** axenfeld-rieger syndrome, glaucoma diagnosis and management, macular edema, masquerade, secondary glaucoma

## Abstract

Axenfeld-Rieger anomaly is a rare, congenital disorder characterized by neural crest-derived defects of the ocular anterior segment, traditionally presenting with high bilateral symmetry. This case report highlights a significant diagnostic pitfall involving a profoundly asymmetric presentation of bilateral Axenfeld-Rieger anomaly in a systemically healthy 23-year-old male student, complicated by late-onset secondary congenital glaucoma and an associated cystoid macular edema. Due to severe, unilateral microcystic epithelial and stromal corneal edema confined strictly to the left eye, the underlying congenital structural anomalies were heavily masked. This advanced unilateral decompensation led two independent tertiary institutions to misdiagnose the condition as an acquired, strictly unilateral iridocorneal endothelial (ICE) syndrome. Meticulous bilateral evaluation resolved the diagnostic dilemma. While the right eye was completely asymptomatic, with normal visual parameters, gonioscopy unmasked silent, subclinical congenital angle anomalies, including an anteriorly inserted iris root and isolated iridocorneal strands distributed across all four quadrants. Crucially, bilateral specular microscopy revealed an absence of pathognomonic ICE cell changes in both eyes, establishing the true bilateral, developmental nature of the pathology. Initial multi-drug medical intraocular pressure-lowering therapies, including the Rho kinase inhibitor ripasudil 0.4%, failed to reverse the advanced left corneal endothelial failure, ultimately necessitating a successful left penetrating keratoplasty for visual rehabilitation. This case underscores the clinical necessity of routine, thorough bilateral gonioscopic and specular evaluations of the clinically unaffected companion eye to avoid diagnostic errors when a young adult presents with sudden, highly asymmetric glaucoma.

## Introduction

Axenfeld-Rieger anomaly is a rare congenital disorder characterized by defective morphogenesis of neural crest-derived mesenchymal cells contributing to the development of the anterior segment of the eye [1]. While the broader term "Axenfeld-Rieger syndrome" encompasses a spectrum of multi-system defects, including dental hypodontia, craniofacial abnormalities, and redundant periumbilical skin, the term "Axenfeld-Rieger anomaly" is used specifically when the developmental tissue alterations are isolated strictly to the ocular tissues without systemic manifestations. The characteristic intraocular phenotype includes an anteriorly displaced, prominent Schwalbe's line (posterior embryotoxon), with tissue strands bridging from the peripheral iris to the trabecular meshwork, and accompanying iris stromal changes such as hypoplasia, corectopia, or polycoria [2]. While the structural angle anomalies are congenital, the development of secondary open-angle glaucoma is reported in approximately 50% of cases, typically manifesting as a delayed complication in early childhood or young adulthood due to the progressive functional compromise of an incompletely developed trabecular meshwork [3].

The clinical expression of Axenfeld-Rieger anomaly is characteristically bilateral; however, the degree of interocular symmetry can vary, and rare exceptions demonstrate substantial interocular phenotypic variability and asymmetry [3]. When marked structural and clinical asymmetry occurs, it can severely obscure the correct diagnosis, leading to clinical dilemmas where the advanced pathology in the symptomatic eye is easily misidentified as an acquired unilateral condition [4]. Furthermore, the unusual co-occurrence of independent retinal pathologies, such as cystoid macular edema (CME), in an eye suffering from advanced anterior segment dysgenesis is poorly documented in current literature, adding another layer of diagnostic complexity. This case report highlights a deceptively asymmetric bilateral presentation of Axenfeld-Rieger anomaly in a systemically healthy 23-year-old male, which was compounded by chronic corneal edema and initially misdiagnosed twice as iridocorneal endothelial (ICE) syndrome.

## Case presentation

A 23-year-old male student presented to our outpatient clinic with a primary complaint of progressive diminution of vision in his left eye spanning 8 months, which was gradual in onset and progressive in nature. The visual decline was accompanied by the perception of colored halos for approximately seven months. The patient's chronological timeline was notable for an episode of metamorphopsia three months prior to presentation, during which external optical coherence tomography (OCT) of the macula documented left-eye cystoid macular edema (CME) of undetermined etiology. He denied any history of ocular trauma, pain, redness, floaters, flashes of light, or limitations in eye movement. Systemic and neurological symptoms, including dental abnormalities, joint hypermobility, walking difficulties, or abdominal wall defects, were entirely absent, establishing the diagnosis of an isolated ocular anomaly rather than a systemic syndrome. Family history indicated that his father exhibited repetitive eye movements, though there was no known family history of glaucoma or blinding ocular diseases.

A comprehensive physical examination and routine multi-specialty clearances from the nephrology, neurology, and cardiology departments were completely unremarkable. Ocular examination demonstrated normal head posture and preserved facial symmetry. Unaided visual acuity in the right eye was 6/6 with no further lens acceptance. In contrast, the left eye demonstrated severely diminished visual acuity to finger counting at a distance of one-half meter, with no improvement with pinhole testing or further refraction.

**Figure 1:**
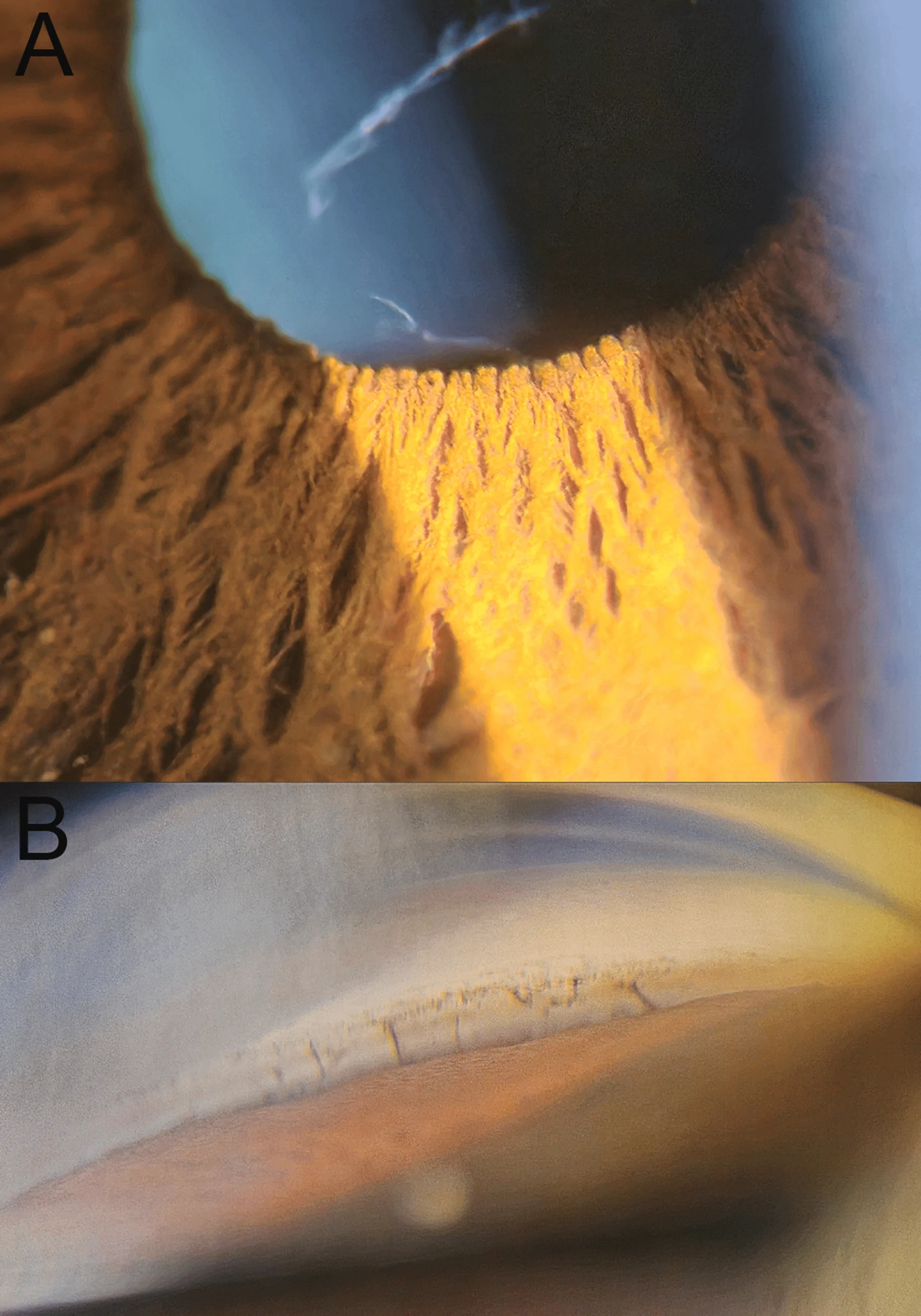
Clinical evaluation and subclinical angle architecture of the companion right eye (A) High-magnification slit-lamp biomicroscopy photograph detailing a clear cornea and the presence of a suspected congenital pupillary membrane along the pupillary border. (B) Gonioscopic photograph of the anterior chamber angle confirming subclinical developmental defects, including localized iridocorneal strands bridging to the Schwalbe line and an anteriorly inserted iris root.

Slit-lamp biomicroscopy of the completely asymptomatic right eye revealed normal lids and adnexa, and a clear cornea with a diameter of 12.0 mm. The right anterior chamber was clear, though a suspected congenital pupillary membrane was visible along the pupillary border (Figure 1A). Right-eye gonioscopy unmasked subclinical, silent defects, revealing localized iridocorneal strands bridging the angle structures distributed across all four quadrants and an anteriorly inserted iris root, alongside a Shaffer's angle grading of two to three (Figure 1B). Crucially, no peripheral anterior synechiae (PAS) were observed in any quadrant of the right eye. Direct ophthalmoscopy of the right eye showed a vertical cup-disc ratio of 0.2:1 with a healthy neuroretinal rim. Intraocular pressure (IOP) measured by non-contact tonometry in the right eye was 20 mmHg.

**Figure 2:**
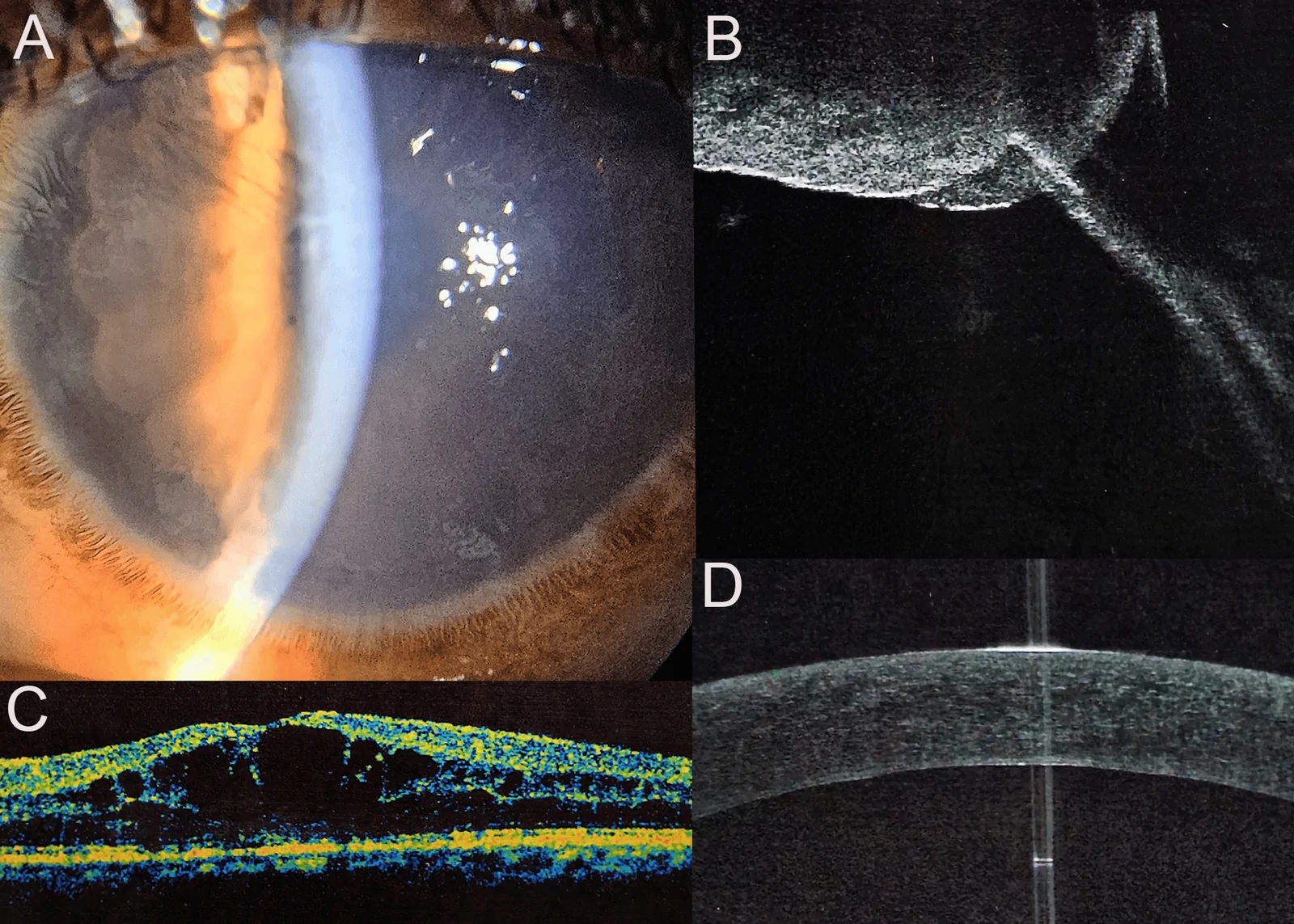
Advanced diagnostic imaging and clinical presentation of the affected left eye (A) Slit-lamp biomicroscopy photograph showing a prominent 360-degree posterior embryotoxon with peripheral corneal structural variations under focal illumination. (B) Anterior segment optical coherence tomography (ASOCT) cross-section confirming the presence of the prominent posterior embryotoxon and peripheral iridocorneal configuration. (C) Optical coherence tomography (OCT) scan demonstrating significant structural fluid collection indicative of associated cystoid macular edema. (D) Anterior segment optical coherence tomography (ASOCT) raw structural scan mapping extensive microcystic changes and established corneal stromal edema. mm = millimeters, mmHg = millimeters of mercury, ASOCT = anterior segment optical coherence tomography, OCT = optical coherence tomography

Examination of the left eye revealed marked microcystic epithelial and established stromal corneal edema, which reduced the tear film break-up time to eight seconds. The left corneal diameter was 12.0 mm. Slit-lamp evaluation of the left anterior chamber revealed a prominent 360-degree posterior embryotoxon and peripheral iridocorneal contact spanning the entire circumference (Figure 2A). The crystalline lens appeared clear bilaterally. The initial intraocular pressure of the left eye was highly elevated at 34 mmHg. Advanced diagnostic imaging via anterior segment optical coherence tomography (ASOCT) confirmed peripheral iridocorneal contact (Figure 2B). Due to the dense corneal edema, the foveal reflex was poorly visible; however, there was documented cystoid macular edema (Figure 2C). Pachymetry verified significant stromal thickening (Figure 2D).

Due to the severe, asymmetric corneal failure confined strictly to the left eye, the underlying congenital structural anomalies were heavily masked, and the patient had been misdiagnosed as having ICE syndrome at two separate tertiary institutions. To resolve this diagnostic dilemma, bilateral specular microscopy was performed, which revealed an absence of the dark-light inversion pattern and pathognomonic ICE cells in either eye, effectively ruling out ICE syndrome and supporting the diagnosis of an asymmetric, bilateral Axenfeld-Rieger anomaly.

The patient was immediately started on maximum medical intraocular pressure-lowering therapy, consisting of topical combination dorzolamide 2% and timolol 0.5% twice daily, topical brimonidine 0.2% twice daily, and the addition of the topical Rho kinase (ROCK) inhibitor ripasudil 0.4% twice daily. Although this comprehensive medical regimen successfully reduced the left eye's intraocular pressure from 34 mmHg to 22 mmHg, the long-standing, irreversible corneal endothelial decompensation was unable to clear. Consequently, a left penetrating keratoplasty (PKP) was performed for visual rehabilitation. Postoperatively, at one month, the corneal graft remained clear, the intraocular pressure remained stable at 18 mmHg on topical medications, and the patient's best-corrected visual acuity improved to 6/36 at the one-month follow-up visit. Long-term management plans include continuous monitoring of central corneal thickness, repeat OCT of the macula to track the resolution of the associated CME, and genetic counseling.

## Discussion

The primary clinical importance of this case lies in its profound interocular phenotypic asymmetry, which presents a significant diagnostic challenge. Due to the severe microcystic epithelial and established stromal corneal edema confined strictly to the left eye, the underlying congenital structural anomalies were heavily masked. This localized unilateral decompensation and elevated IOP led to the case being misdiagnosed at two independent tertiary institutions as ICE syndrome--specifically Chandler’s syndrome--which classic literature defines as an acquired, progressive, and characteristically unilateral disease entity [5].

Differentiating between Axenfeld-Rieger anomaly and ICE syndrome relies heavily on a meticulous bilateral gonioscopic evaluation. While the patient’s right eye was completely asymptomatic with normal visual parameters, gonioscopy unmasked silent, subclinical congenital alterations, including localized iridocorneal strands distributed across all four quadrants and an anteriorly inserted iris root [3]. This finding of fellow-eye abnormalities is a critical diagnostic indicator, as a completely normal companion eye is expected in true ICE syndrome, whereas misdiagnosed bilateral ICE presentations are often unmasked as developmental anomalies [4]. Bilateral specular microscopy further refined the clinical assessment; rather than confirming an Axenfeld-Rieger phenotype directly, its primary value lay in verifying the absence of the dark-light cell inversion pattern and pathognomonic 'ICE cells' characteristic of iridocorneal endothelial changes [4,5]. The delayed onset of secondary glaucoma at 23 years of age illustrates that while the developmental angle anomalies are present at birth, progressive functional outflow compromise can remain clinically silent until adulthood [3].

The chronological association with CME in this case represents an associated finding of uncertain significance. Although a mechanical or vascular cascade driven by subclinical intraocular pressure elevations altering the blood-retinal barrier could be hypothesized, the available clinical data demonstrate a temporal association rather than a definitive causal relationship. Furthermore, the management of this case underscores that glaucoma filtration surgery or maximum medical therapy, including the combination dorzolamide/timolol, brimonidine, and the Rho kinase (ROCK) inhibitor ripasudil 0.4%, is intended primarily for strict IOP control to prevent progressive glaucomatous optic atrophy, rather than directly clearing the corneal haze [3]. When maximum medical therapy failed to clear the irreversible corneal endothelial failure despite optimizing the IOP, a left penetrating keratoplasty (PKP) was required for visual rehabilitation.

Finally, due to the congenital nature of this anomaly and the history of eye movement abnormalities in the patient’s father, multi-generational genetic counseling and molecular profiling remain strongly indicated [6]. However, an autosomal dominant inheritance pattern cannot be definitively confirmed in this patient without formal genetic testing. Ultimately, this case delivers a clear clinical message that marked interocular asymmetry does not exclude bilateral anterior segment dysgenesis, and a thorough examination of the clinically unaffected fellow eye is absolutely mandatory whenever ICE syndrome is suspected.

## Conclusions

This case underscores that markedly asymmetric bilateral Axenfeld-Rieger anomaly can closely mimic an acquired unilateral condition like iridocorneal endothelial (ICE) syndrome, presenting a distinct diagnostic pitfall in young adults. A meticulous bilateral gonioscopic and specular microscopic evaluation remains the principal clinical tool in these scenarios, as unmasking silent, subclinical developmental angle defects in the completely asymptomatic companion eye is key to establishing the correct bilateral diagnosis. Additionally, the co-occurrence of cystoid macular edema (CME) must be recognized as an associated finding of uncertain significance rather than as a proven mechanistic consequence of anterior segment dysgenesis affecting intraocular dynamics or the blood-retinal barrier. Ultimately, severe clinical asymmetry does not exclude bilateral congenital anomalies, and careful examination of the fellow eye is mandatory whenever a unilateral endothelial or syndromic process is suspected.
